# Supplementary material for: Identification of Pri-miRNA Stem-Loop Interacting Proteins in Plants Using a Modified Version of the Csy4 CRISPR Endonuclease
Source: Int J Mol Sci. 2022 Aug 11;23(16):8961. doi: 10.3390/ijms23168961 (PMC9409100; doi:10.3390/ijms23168961)
Supplement: Supplementary file 1 [file ijms-23-08961-s001.zip › Supplementary Materials.pdf]

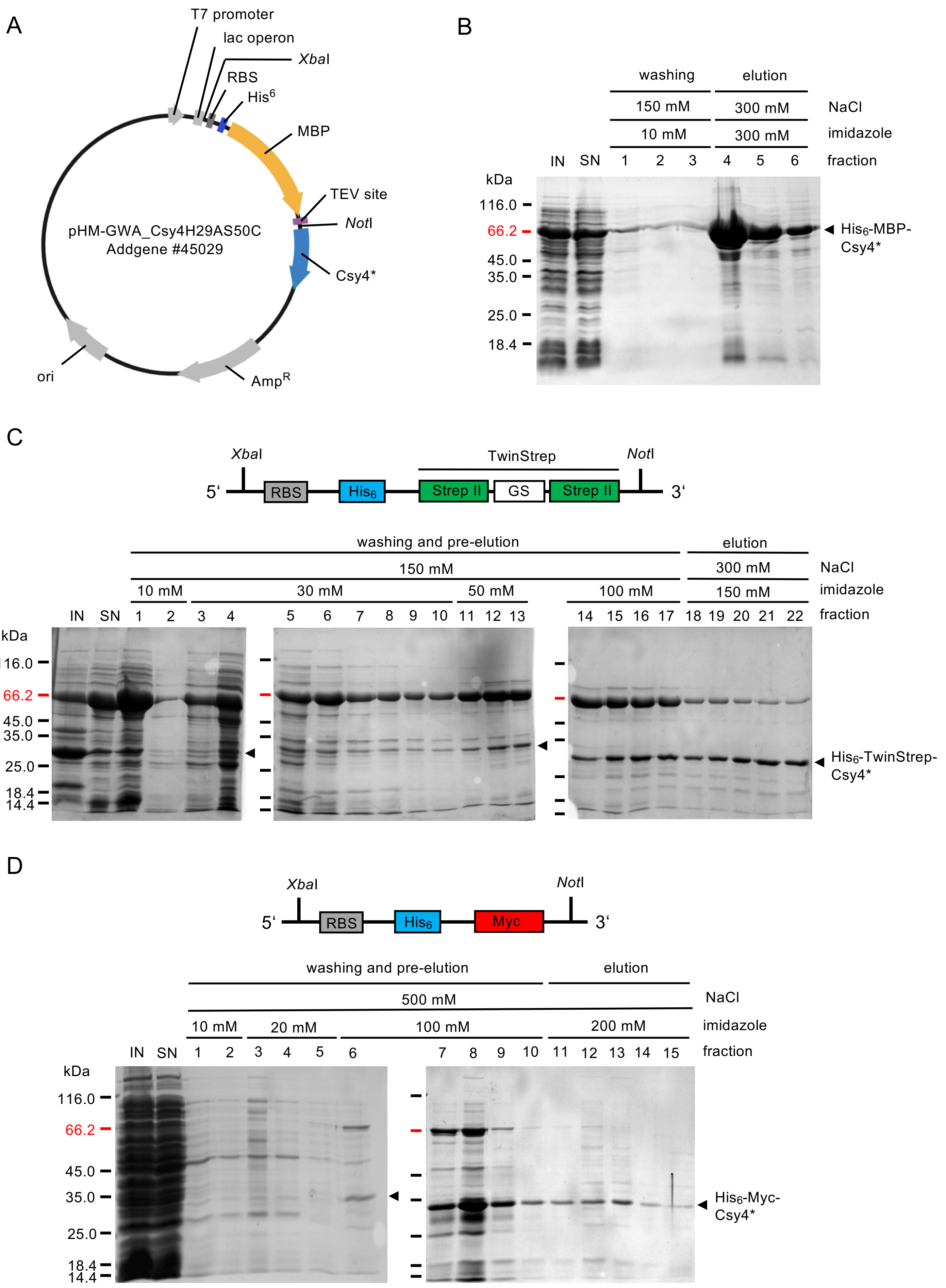

A
T7 promoter
lac operon
XbaI
RBS
His6
MBP
TEV site
NotI
Csy4*
pHM-GWA_Csy4H29AS50C
Addgene #45029
ori
AmpR
B
washing
elution
150 mM
300 mM
NaCl
10 mM
300 mM
imidazole
IN SN 1 2 3 4 5 6
fraction
kDa
116.0
66.2
45.0
35.0
25.0
18.4
His6-MBP-Csy4*
C
XbaI
TwinStrep
NotI
5‘
RBS
His6
Strep II
GS
Strep II
3‘
washing and pre-elution
elution
150 mM
300 mM
NaCl
10 mM
30 mM
50 mM
100 mM
150 mM
imidazole
IN SN 1 2 3 4 5 6 7 8 9 10 11 12 13 14 15 16 17 18 19 20 21 22
fraction
kDa
116.0
66.2
45.0
35.0
25.0
18.4
14.4
His6-TwinStrep-Csy4*
D
XbaI
NotI
5‘
RBS
His6
Myc
3‘
washing and pre-elution
elution
500 mM
NaCl
10 mM
20 mM
100 mM
200 mM
imidazole
IN SN 1 2 3 4 5 6 7 8 9 10 11 12 13 14 15
fraction
kDa
116.0
66.2
45.0
35.0
25.0
18.4
14.4
His6-Myc-Csy4*

Figure S1: Recombinant expression and purification of Csy4* protein variants. (A) Depiction of the pHM-GWA_Csy4H29AS50C vector (Addgene #45029) used for recombinant expression of His_6-MBP-Csy4*. Recombinant expression of His_6-MBP-Csy4* (B), His_6-TwinStrep-Csy4* (C) and His_6-Myc-Csy4* (D). Top panels in C and D represent the sequence regions replacing His_6-MBP in pHM-GWA_Csy4H29AS50C. Protein samples were fractionated on SDS-PAGE gels and visualized by coommassie staining. IN, input (total protein extract). SN, supernatant after immunopurification.

A

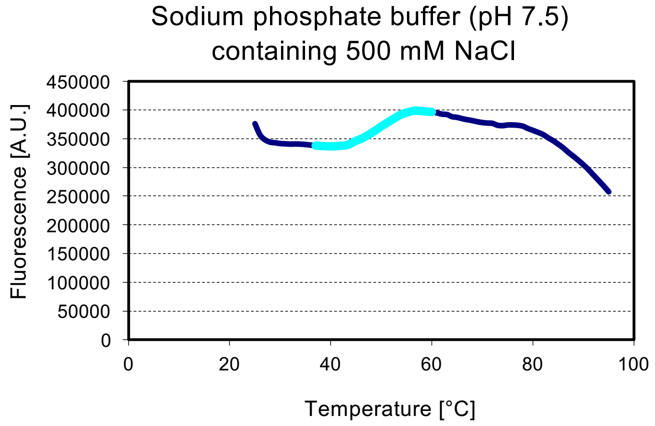

B

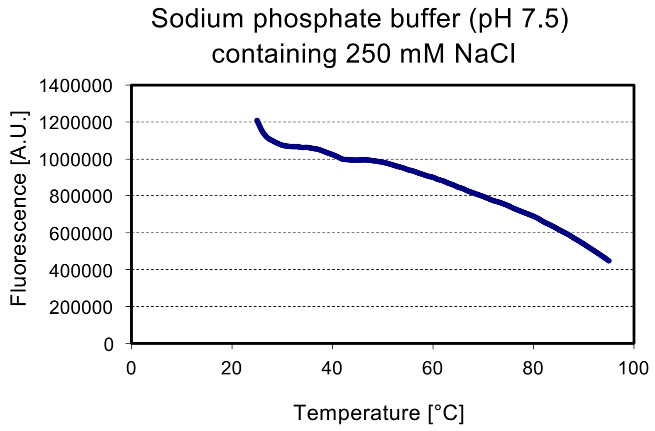

Figure S2: Measuring the stability of His$_6$-Myc-Csy4* protein by differential scanning fluorimetry (DSF). Graphs showing recording of fluorescence intensity versus temperature for His$_6$-Myc-Csy4* in sodium phosphate buffer (pH 7.5) containing 500 mM (A) or 250 mM NaCl (B). Protein unfolding is measured by an increase in the fluorescence of the dye SYPRO orange with affinity for hydrophbic parts of the protein, which are exposed as the protein unfolds. A sigmoidal increase in fluorescence to a plateau with gradually increasing temperature is desired and highlighted in light blue [25].

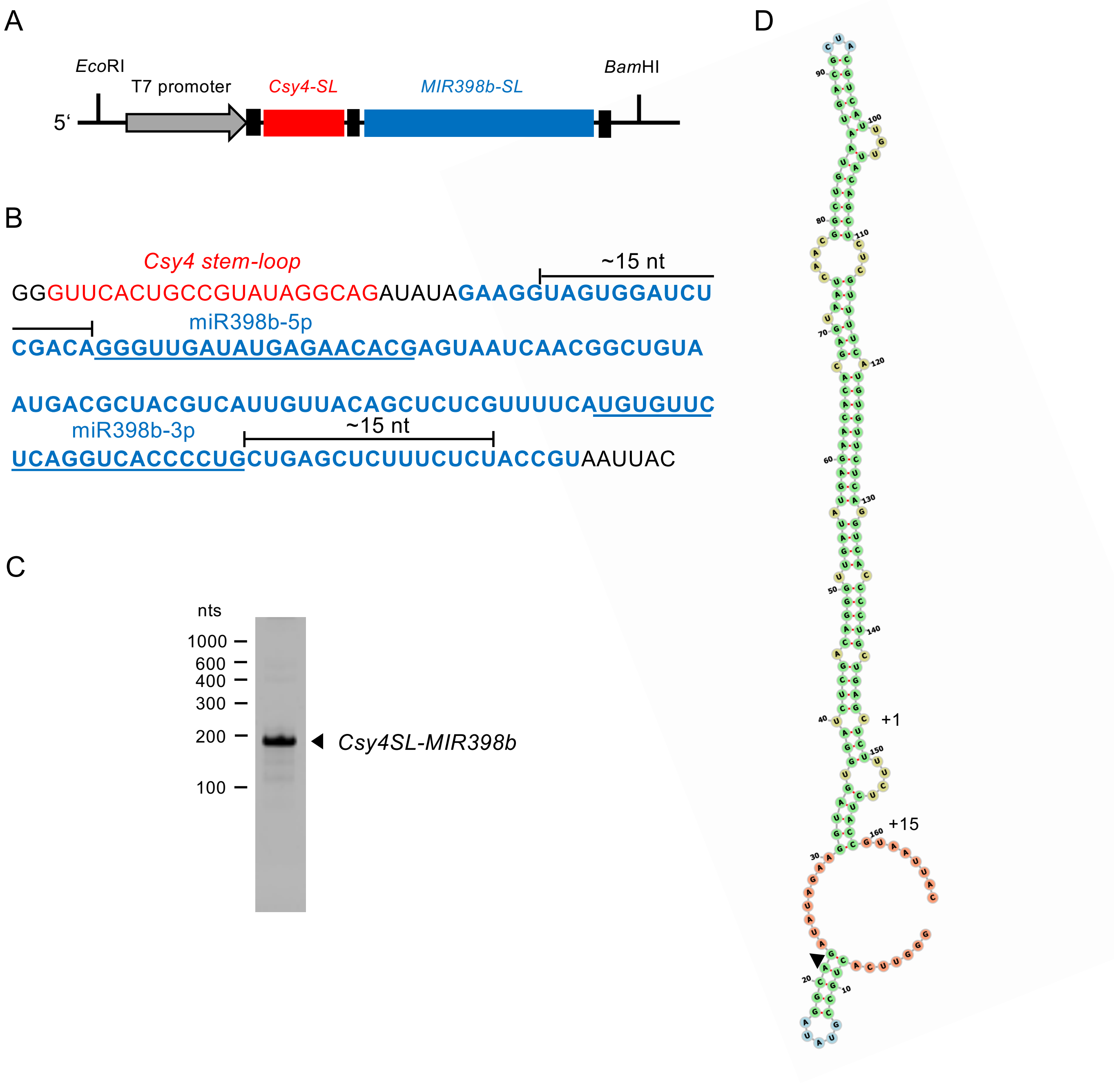

Figure S3: Design and *in vitro* transcription of the tagged pri-miR398b stem-loop. (A) Schematic representation of the construct used for *in vitro* transcription of the Csy4-hairpin tagged pri-miR398b stem-loop. (B) Sequence of the *in vitro* transcribed Csy4-hairpin tagged pri-miR398b stem-loop. (C) Visualization of the *in vitro* transcribed Csy4-hairpin tagged pri-miR398b stem-loop (*Csy4SL-MIR398b*) by urea-PAGE. (D) Secondary structure prediction of the Csy4-hairpin tagged pri-miR398b stem-loop using RNAfold from the Vienna RNA Websuite.

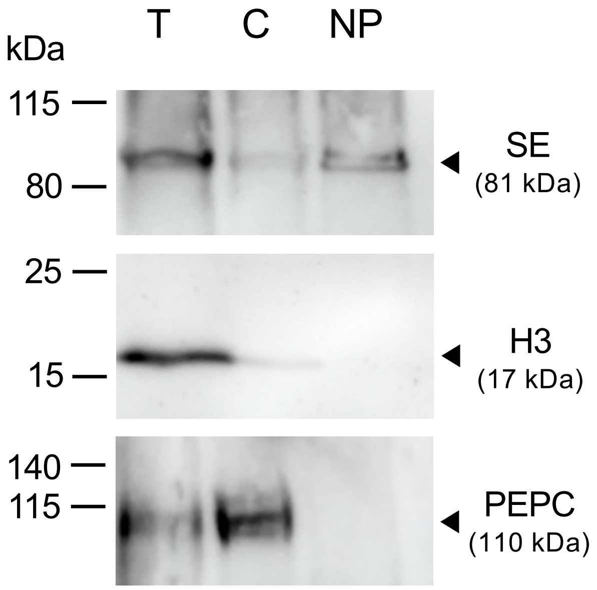

Figure S4: Purity evaluation of the nucleoplasmic extracts. Immunoblot analysis was performed with antibodies against specific markers for the nucleoplasm (SERRATE, SE), the chromatin (HISTONE 3, H3), and the cytoplasm (PHOSPHOENOLPYRUVATE CARBOXYLASE, PEPC). T, total protein. C, cytoplasmic fraction. NP, nucleoplasmic fraction.

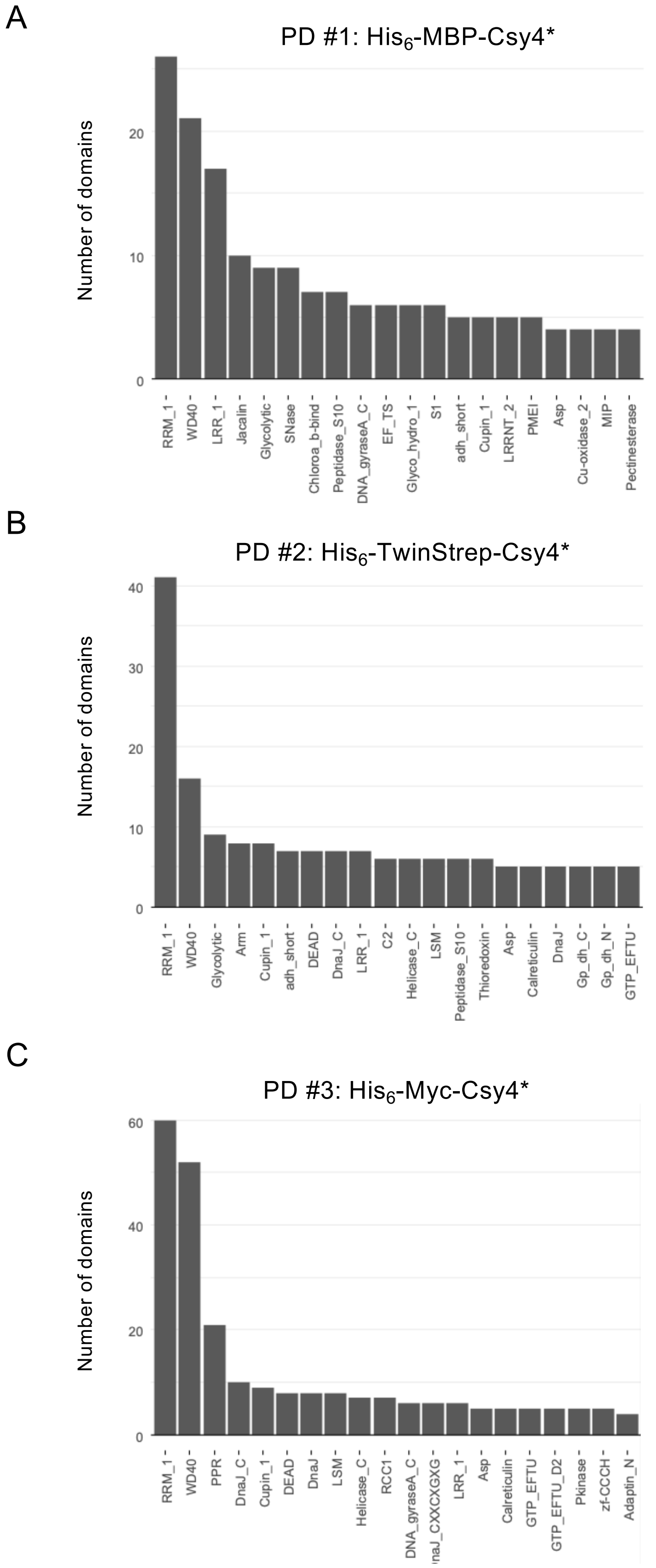

Figure S5: Top 20 protein domains recognized among co-purified proteins. Number of domains identified from the His_6-MBP-Csy4* (A), His_6-TwinStrep-Csy4* (B) or His_6-Myc-Csy4* RNA pulldown (C).
